# *Tropheryma whipplei* Endocarditis

**DOI:** 10.3201/eid1911.121356

**Published:** 2013-11

**Authors:** Florence Fenollar, Marie Célard, Jean-Christophe Lagier, Hubert Lepidi, Pierre-Edouard Fournier, Didier Raoult

**Affiliations:** Aix-Marseille Université, Marseille, France (F. Fenollar, J.-C. Lagier, H. Lepidi, P.-E. Fournier, D. Raoult);; Assistance Publique Hopitaux de Marseille, Marseille (F. Fenollar, J.-C. Lagier, P.-E. Fournier, D. Raoult);; Groupement Hospitalier Est, Bron, France (M. Célard)

**Keywords:** Whipple’s disease, Whipple disease, *Tropheryma whipplei*, endocarditis, arthralgia, bacteria

## Abstract

*Tropheryma whipplei* endocarditis differs from classic Whipple disease, which primarily affects the gastrointestinal system. We diagnosed 28 cases of *T. whipplei* endocarditis in Marseille, France, and compared them with cases reported in the literature. Specimens were analyzed mostly by molecular and histologic techniques. Duke criteria were ineffective for diagnosis before heart valve analysis. The disease occurred in men 40–80 years of age, of whom 21 (75%) had arthralgia (75%); 9 (32%) had valvular disease and 11 (39%) had fever. Clinical manifestations were predominantly cardiologic. Treatment with doxycycline and hydroxychloroquine for at least 12 months was successful. The cases we diagnosed differed from those reported from Germany, in which arthralgias were less common and previous valve lesions more common. A strong geographic specificity for this disease is found mainly in eastern-central France, Switzerland, and Germany. *T. whipplei* endocarditis is an emerging clinical entity observed in middle-aged and older men with arthralgia.

Whipple disease was first described in 1907 (*1*). This chronic infection is characterized by histologic indication of gastrointestinal involvement, determined by a positive periodic acid–Schiff (PAS) reaction in macrophages from a small bowel biopsy sample (*2*). It is caused by *Tropheryma whipplei* and encompasses asymptomatic carriage of the organism to a wide spectrum of clinical pathologic conditions, including acute and chronic infections (*1*,*2*).

In 1997, *T. whipplei* was first implicated as an agent of blood culture–negative endocarditis in 1 patient by use of broad-range PCR amplification and direct sequencing of 16S rRNA applied to heart valves from patients in Switzerland (*3*). Two years later, 4 additional cases were reported in Switzerland (*4*). In 2000, the first strain of *T. whipplei* was obtained from the aortic valve of a patient with blood culture–negative endocarditis (*5*). 

Blood culture–negative endocarditis accounts for 2.5%–31.0% of all cases of endocarditis. The incidence rate of *T. whipplei* endocarditis among blood culture–negative endocarditis cases has not been established; however, at our center (Assistance Publique Hôpitaux de Marseille, Marseille, France), this incidence rate was estimated to be 2.6% (*6*). In Germany, the reported incidence rate for *T. whipplei* endocarditis is 6.3%: *T. whipplei* was the fourth most frequent pathogen found among 255 cases of endocarditis with an etiologic diagnosis and was the most common pathogen associated with blood culture–negative endocarditis. This incidence rate exceeds rates of infections caused by *Bartonella Quintana*; *Coxiella burnetii*; and members of the *Haemophilus*, *Actinobacillus*, *Cardiobacterium, Eikenella*, *Kingella* spp. group (*7*). Smaller studies found incidence rates of 3.5% in Denmark (*8*), 4.3% in Switzerland (*9*), 7.1% in the Czech Republic (*10*), 2.8% in Spain (*11*), and none in Algeria (*12*). We describe 28 cases of *T. whipplei* endocarditis and compare them with cases reported in the literature.

## Materials and Methods

### Patient Recruitment and Case Definitions

Our center in Marseille, France, has become a referral center for patients with *T. whipplei* infections and blood culture–negative endocarditis (*2*,*5*,*6*). We receive samples from France and other countries. Each sample is accompanied by a questionnaire, completed by the physician, covering clinico-epidemiologic, biological, and therapeutic data for each patient. We analyzed data from October 2001 through April 2013. Diagnosis of *T. whipplei* endocarditis was confirmed by positive results from PAS staining and/or specific immunohistochemical analysis and 2 positive results from specific PCRs of a heart valve specimen in addition to lack of histologic lesions in small bowel biopsy samples or lack of clinical involvement of the gastrointestinal tract.

### Laboratory Procedures

DNA was extracted from heart valves, 200 µL of body fluid (blood in a tube containing EDTA, saliva, or cerebrospinal fluid), small bowel biopsy samples, and ≈1 gram of feces by using QIAGEN columns (QIAamp DNA kit; QIAGEN, Hilden, Germany) according to the manufacturer’s recommendations. Quantitative real-time PCR (qPCR) was performed by using a LightCycler instrument (Roche Diagnostics, Meylan, France) and the QuantiTect Probe PCR Kit (QIAGEN) according to the manufacturer’s guidelines. From October 2001 through September 2003, all specimens were tested by qPCR selective for the 16S–23S rRNA intergenic spacer and the *rpoB* gene, as described (*13*); from October 2003 through March 2004, all specimens were tested by qPCR selective for *T. whipplei* repeated sequences (repeat PCR), as described (*13*). When an amplified product was detected, sequencing was systematically performed. Since April 2004, all specimens have been tested by a qPCR selective for *T. whipplei* repeated sequences, which used specific oligonucleotide Taqman probes for the identification (*13*). To validate the tests, we used positive and negative controls (*13*). For determination of DNA extract quality, the human actin gene was also detected. For positive specimens, *T. whipplei* genotyping was performed as described (*14*). In parallel, all heart valves and blood samples from patients with suspected endocarditis underwent systematic PCR screening for all bacteria (16S rRNA) and all fungi (18S rRNA) and underwent specific real-time PCR selective for *Streptococcus oralis* group, *Streptococcus gallolyticus* group, *Enterococcus faecium* and *E. faecalis*, *Staphylococcus aureus*, *Mycoplasma* spp., *Coxiella burnetii*, *Bartonella* spp., and *T. whipplei* as described (*6*).

For histologic analysis, formalin-fixed paraffin-embedded heart valves and small bowel biopsy samples were cut in thin sections. Samples stained with hematoxylin-eosin-saffron and special stains were examined, and immunohistochemical investigations with a specific antibody were performed as reported (*15*). Cardiac valve and heparinized blood specimens were injected into cell and axenic cultures (*5*,*16*). Serologic assays were based on Western blot analyses (*17*).

### Statistical Analysis

Statistical analyses were performed by using EpiInfo6 (www.cdc.gov/epiinfo/Epi6/EI6dnjp.htm). A p value <0.05 was considered significant. Data for the population of France were extracted from the National Institute for Statistics and Economical Studies website (www.insee.fr/fr/).

### Literature Review

We searched the PubMed database (www.ncbi.nlm.nih.gov/pubmed/) through April 2013, using the keywords “Tropheryma,” “Whipple’s disease,” and “endocarditis.” We then performed a cross-reference analysis on the results of the search. For this literature review, the patient inclusion criteria were lack of histologic evidence of small bowel involvement or lack of diarrhea.

## Results

### Epidemiologic and Clinical Characteristics

Among the 28 patients for whom data enabled the diagnosis of *T. whipplei* endocarditis; 16 have been previously reported or cited (*2*,*5*,*18*,*19*). According to current modified Duke criteria (*20*) before the examination of heart valve specimens, only 1 (3.6%) patient met the criteria for endocarditis and 17 (60.7%) met the criteria for possible endocarditis (Technical Appendix Table 1)

**Figure 1:**
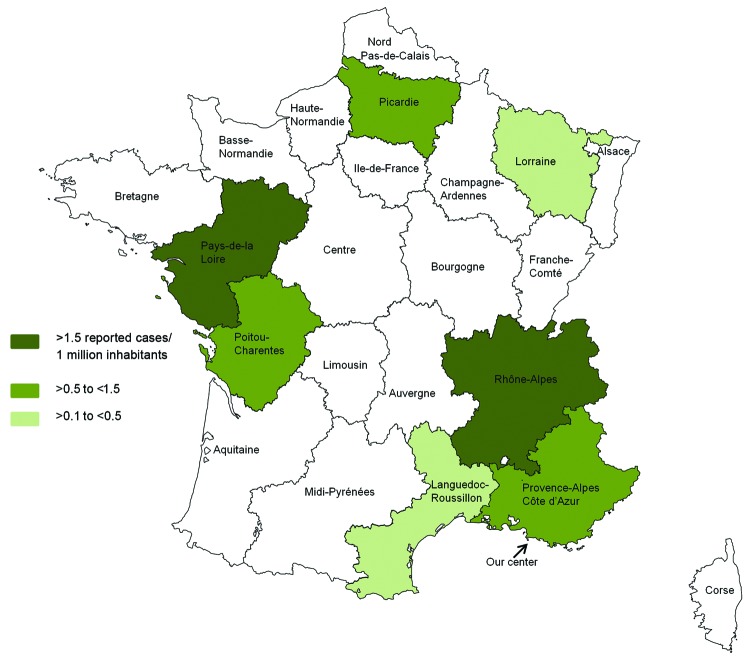
Number of reported cases of *Tropheryma whipplei* endocarditis per 1 million inhabitants in each area of France over 10 years. Data from this series and the literature (*22**–**24*) were included. Among the metropolitan areas in France, the incidence of *T. whipplei* endocarditis is significantly more frequent in the Rhône-Alpes area than in 11 others areas (Alsace, Aquitaine, Basse-Normandie, Bourgogne, Centre, Champagne-Ardenne, Haute-Normandie, Ile de France, Languedoc-Roussillon, Midi-Pyrénées, and Nord Pas-de-Calais; p = 0.04, p = 0.004, p = 0.048, p = 0.04, p = 0.01, p = 0.04, p = 0.02, p<0.001, p = 0.04, p = 0.007, p = 0.006, respectively). The incidence rate is also significantly more frequent in the Pays de la Loire area than in 6 other areas (Aquitaine, Bretagne, Centre, Ile-de France, Lorraine, Midi-Pyrénées, Nord Pas de Calais; p = 0.04, p = 0.04, p = 0.04, p = 0.003, p = 0.03, p = 0.02, respectively).

All 28 patients underwent heart valve replacement because of valve damage (*21*). All patients were male (Table 1), and mean age (± SD) was 58.6 (± 10) years (range 40–80 years). Among the 27 patients from France, 13 (48.1%) were living in the Rhône-Alpes area and 5 (18.5%) in the Pays de la Loire area. Although samples are sent from all over France, 66.6% of the patients were from only these 2 areas. If we focus on the 702 patients with blood culture–negative endocarditis in France referred to our center from May 2001 through September 2009 (Technical Appendix Table 2), the patients from these 2 areas were significantly more affected than the rest of the population (Rhône-Alpes 9/106 [8.5%] vs 7/596 [1.16%], p<0.001 and Pays de la Loire 4/26 [15.4%] vs 12/676; p<0.001) (*6*). Incidence rates of *T. whipplei* endocarditis in these 2 areas are also significantly higher than those in the rest of France (Rhône-Alpes 0.25 cases/1 million inhabitants/year, p<0.001; Pays de la Loire (0.15 cases/1 million inhabitants/year, p<0.001) (Figure 1).

**Table 1:** Epidemiologic and clinical characteristics of 28 patients with *Tropheryma whipplei* endocarditis*

Patient (reference)	Area of origin	Age, y	IS	Cardiac history	Arthralgia duration, y	Weight loss	Cardiac symptoms	Fever	Involved valves	Vegetation
1	PACA	80	N	PM	Y (15)	N	HF and AIS	N	AV	Y
2	Corsica†	58	N	N	Y (8)	N	HF	Y	AV+MV	N
3	Rhône-Alpes	54	N	N	Y (3)	Y	HF	Y	AV	Y
4	Rhône-Alpes	45	N	BAV	N	N	Stroke	Y	AV	Y
5	Rhône-Alpes	56	N	CS	N	N	HF	Y	AV	Y
6	PACA	59	Y	N	Y (35)	N	HF	N	AV	N
7	Pays de la Loire	50	N	N	Y (NA)	N	HF	N	AV+MV	Y
8	Picardie	51	N	N	N	N	Stroke	N	AV	Y
9	PACA	79	N	AVB	Y	N	HF	N	AV	Y
10	Lorraine	50	N	N	N	N	HF	N	MV	N
11	Rhône-Alpes	62	N	N	Y	N	HF	N	AV	Y
12	PACA	58	N	BAV	Y	Y	HF	Y	AV	Y
13 *(2)*	Rhône-Alpes	71	N	N	N	N	HF	N	AV	Y
14 *(18)*	Rhône-Alpes	57	Y	N	Y (4)	N	HF+PAE	Y	AV	N
15 *(2)*	Poitou-Charentes	68	N	N	N	N	HF	Y	AV	Y
16 *(2)*	Pays de la Loire	48	N	AVI	Y (NA)	N	HF	Y	AV	Y
17 *(19)*	Languedoc-Roussillon	70	Y	N	Y (27)	N	HF	Y	AV	Y
18 *(2)*	Pays de la Loire	71	N	MVI	Y (NA)	N	HF	Y	MV	Y
19 *(2)*	Pays de la Loire	57	Y	N	Y (10)	Y	HF	Y	AV+MV	N
20 *(2)*	Rhône-Alpes	67	N	N	Y (4)	N	Stroke	N	AV	N
21 *(2)*	Rhône-Alpes	51	Y	N	Y (6)	N	PAE	N	AV+TV	Y
22 *(2)*	Pays de la Loire	58	N	AAR	Y (NA)	N	PAE	N	AV	Y
23 *(2)*	Rhône-Alpes	60	Y	N	Y (2)	N	HF	N	AV+MV	Y
24 *(2)*	Rhône-Alpes	61	N	AAR	Y (4)	N	Stroke	N	AV+MV	Y
25 *(2)*	Rhône-Alpes	63	N	N	Y (3)	N	PAE+stroke	N	AV	Y
26 *(5)*	Canada	42	N	AAR	N	Y	HF	N	AV+MV	Y
27 *(2)*	Rhône-Alpes	40	Y	AAR	Y (5)	N	HF	N	AV	Y
28 *(2)*	Rhône-Alpes	55	N	N	Y (5)	Y	Stroke	N	MV	Y

*All patients were male, and none had diarrhea. IS, immunosuppressive therapy; PACA, Provence-Alpes-Côte-d’Azur; N, no; PM, pacemaker; Y, yes; HF; heart failure; AIS, acute ischemic stroke; AV, aortic valve; MV, mitral valve; BAV, bicuspid aortic valve; CS, coronary stent; AVB, aortic valve bioprosthesis; AVI, aortic valve insufficiency; MVI, mitral valve insufficiency; NA, not available; PAE, peripheral arterial embolism; AAR, acute articular rheumatism. †This patient was from Poland but resided in Corsica for 8 years.

Immunosuppressive therapy had been given to 7 (29%) patients, of which 3 received a tumor necrosis factor inhibitor. Arthralgia was reported for 21 (75%) patients; mean delay between arthralgia onset and endocarditis diagnosis was 8.5 years. Among 12 patients who had been interviewed by 1 of the authors (D.R.), arthralgia was detected in 11. Among these 12, arthralgia was retrospectively noticed by 1 patient who, after beginning treatment for endocarditis, reported the disappearance of slight pain that had been present for many years. Previous heart valve disease was known for 9 (32%) patients. Heart failure occurred in 20 (71.4%) patients, acute ischemic stroke in 7 (29.2%), and peripheral arterial embolism in 4 (16.6%). Fever was detected in 11 (39%) patients, and weight loss was experienced by 4 (14.3%). Echocardiography was performed for all 28 patients: transthoracic echocardiography for 4 patients, transesophageal echocardiography for 9 patients, both procedures for 7 patients, and unspecified procedures for 8 patients. Cardiac vegetations were found in 22 (78.6%) patients, and aortic valve involvement was found in 18 (64.2%).

### Laboratory Findings

At the time of *T. whipplei* endocarditis diagnosis, increased C-reactive protein levels were detected in 17 (81%) of 21 patients, anemia in 6 (37.5%) of 16, and leukocytosis in 5 (29.5%) of 17. *T. whipplei* endocarditis was diagnosed by heart valve analysis (either PCR for *T. whipplei* or histologic analysis) for 27 of the 28 patients (Technical Appendix Table 3). Other molecular analyses were negative for other microorganisms on all heart valves and in blood samples (when available). Blood samples were positive for *T. whipplei* for 5 (31.2%) of 16 patients. For 1 patient (patient 12), at 4 days before heart valve replacement, a blood sample was positive for *T. whipplei* according to repeat PCR and negative according to 16S rRNA PCR. For another patient (patient 1), a pacemaker was positive for *T. whipplei* by PCR. *T. whipplei* was detected in 1 (6.7%) of 15 saliva samples, 2 (16.6%) of 12 fecal samples, and none of 8 cerebrospinal fluid samples.

**Figure 2:**
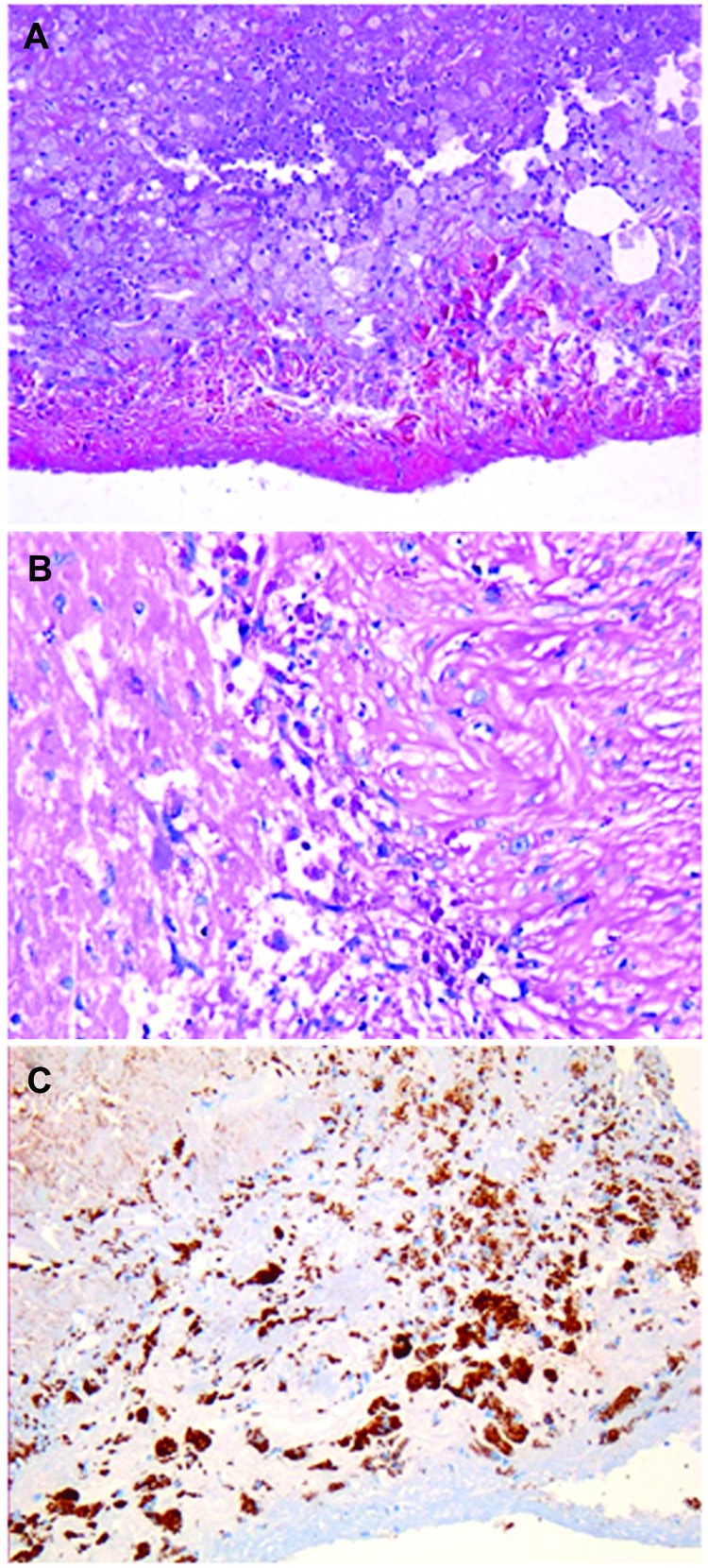
Aortic valve from patient with *Tropheryma whipplei* endocarditis. A) Hematoxylin–eosin–saffron stain (original magnification ×100). B) Foamy macrophages containing characteristic inclusion bodies (periodic acid–Schiff stain; original magnification ×200). C) Immunostaining of *T. whipplei* with polyclonal rabbit antibody against *T. whipplei* and Mayer’s hemalum counterstain (original magnification ×100). No destruction of this valve is visible.

*T. whipplei*–infected heart valves show the typical histologic features of infective endocarditis: vegetations, inflammatory infiltrates, and valvular destruction (*15*). They show fibrotic, scarred areas. Valvular inflammatory infiltrates mainly consisted of foamy macrophages and lymphocytes. The foamy histiocytes were filled with dense and granular material that was strongly positive on PAS staining and resistant to diastase or immunopositive with a specific antibody against *T. whipplei*. *T. whipplei*–infected macrophages were seen in the vegetations on the surface of the heart valves and more deeply in the valvular tissues (Figure 2). An arterial embolus surgically removed from the lower limb of patient 22 was positive by immunodetection (*15*). However, 1 year before the heart valve was removed, this embolus had been histologically analyzed but infection was not suspected. Only after the valve was found to be positive for *T. whipplei* did subsequent analyses show that the embolus was positive for *T. whipplei*. Small bowel biopsy samples were obtained for 19 patients; all samples were negative by PAS staining, probably ruling out asymptomatic involvement of the gastrointestinal tract.

Two strains of *T. whipplei* were isolated from blood specimens, and 7 strains (including the strain from the index case-patient) from heart valve culture (*5*). For patient 20, a strain was isolated from the blood and heart valve specimens. The delay in primary isolation was 2 weeks for the heart valve sample and 8 weeks for the blood sample. No other microorganism was isolated. Serum was available for 18 patients. According to our previously established criteria, 10 (55.5%) patients had a negative or weakly positive serologic profile, as is observed for patients with classic Whipple disease, and 8 (44.5%) patients had a frankly positive profile, as is observed for chronic carriers. This finding suggests a potentially less decreased antibody-mediated immune response for these patients (*17*). Thus, the previously established serologic profile for patients with classic Whipple disease is observed significantly less frequently among patients with *T. whipplei* endocarditis (10/18) than among patients with classic Whipple disease (56/60; p<0.001). The serologic profile previously observed for chronic carriers also occurs significantly less frequently among patients with endocarditis (24/26 vs 8/18; p = 0.01).

*T. whipplei* genotype was obtained for 19 heart valves samples. Genotype 3 was detected in 5 samples, and genotype 1 was detected in 2 samples. The other 12 samples harbored a unique genotype. Genotypes for 4 patients were those previously detected in other circumstances (genotypes 8, 11, 19, and 97). Only patients with *T. whipplei* endocarditis had genotypes 7, 24, 87, 90, 96, 99, 113, 117. For 1 of these patients (patient 13), genotype 7 was detected in a heart valve sample at the time of diagnosis in 2002, but genotype 101 was detected in saliva and fecal samples in 2011 (Table 2). The patient did not have characteristics that favor endocarditis relapse.

**Table 2:** Treatment, outcome, and follow-up data for 14 patients with *Tropheryma whipplei* endocarditis managed entirely by our team*

Patient no.†	First drug (duration)	Second drug (duration)†	Outcome	Length of follow-up at the end of the last treatment
1	AMX + GEN (15 d)	DOX + HCQ (ongoing)	Well, including arthralgia disappearance	Ongoing treatment
2	AMX + GEN (15 d)	DOX + HCQ (ongoing)	Well, including arthralgia disappearance	Ongoing treatment
5	CEF + GEN (15 d)	DOX + HCQ (ongoing)	Well	Ongoing treatment
6	NA	DOX + HCQ (1 yr)	Well	1 yr
7	AMX + GEN (NA)	DOX + HCQ (7 mo)	Relapse 4 mo after the end of treatment; prosthetic dehiscence without fever; heart valve positive by PAS and immunohistochemical staining; negative by PCR	Ongoing new treatment
9	AMC + GEN (11 d)	DOX + HCQ (ongoing)	Well	Ongoing treatment
12	CEF (5 d)	DOX + HCQ (ongoing)	Well	Ongoing treatment
14	CEF + GEN (15 d)	DOX + HCQ (1 yr)	Well	2.5 yr
17	CEF + GEN (15 d)	DOX + HCQ (1.5 yr)	Well	3.5 yr
20	NA	DOX + HCQ (1 yr)	Well	6 mo
21	AMX + GEN (18 d)	SXT (1.5 yr)	Well	5 yr
23	VAN + DOX + OFX (19 d)	DOX + HCQ (1.5 yr)	1 yr after end of treatment, saliva sample positive for *T. whipplei* by PCR (genotype NA); SXT started and continued for 12 mo	9 mo after onset of lifelong prophylaxis
			5.5 yr after end of treatment, saliva and fecal samples positive for *T. whipplei* by PCR (new genotype: 101); no cardiac abnormalities observed; started lifelong prophylaxis with DOX at 100 mg 2×/d; well	
24	AMX + GEN (4 wk)	DOX + HCQ (1.5 yr)	Well	2 yr, then colon cancer and death
25	AMX + GEN (15 d)	SXT (14 mo)	12 mo after the end of the treatment: saliva specimen positive for *T. whipplei* by PCR (genotype NA); SXT replaced DOX + HCQ after a perforated sigmoid diverticulitis with spreading peritonitis for 18 mo; well	6 yr

*Team from Assistance Publique Hôpitaux de Marseille, Marseille, France. All patients had undergone heart valve surgery. AMX, amoxicillin; GEN, gentamicin; HCQ,, hydroxychloroquine; PAS, periodic acid–Schiff; CEF, ceftriaxone ; AMC, amoxicillin–clavulanate; VAN, vancomycin; DOX, doxycycline; OFX, ofloxacin. †DOX at 100 mg 2×/d and HCQ at 200 mg 3×/ d; SXT at 320 mg trimethoprim and 1,600 mg sulfamethoxazole 3×/d.

### Treatment and Outcomes

We focused on 14 patients for whom the entire treatment was managed by our team, 13 of whom regularly consulted author D.R. (Table 2). Overall, 12 patients received a combination of doxycycline and hydroxychloroquine for 7–18 months, and 2 received trimethoprim–sulfamethoxazole. One patient who experienced relapse received treatment for 7 months. According to analysis of saliva and fecal samples, 2 patients had been colonized by *T. whipplei* at another time. Colonization of 1 of these patients was with a new strain, but neither had cardiac abnormalities. We prescribed treatment for these patients, including 1 who had been taking lifelong prophylactic doxycycline, as reported for a patient with classic Whipple disease (*25*).

### Literature Review

**Figure 3:**
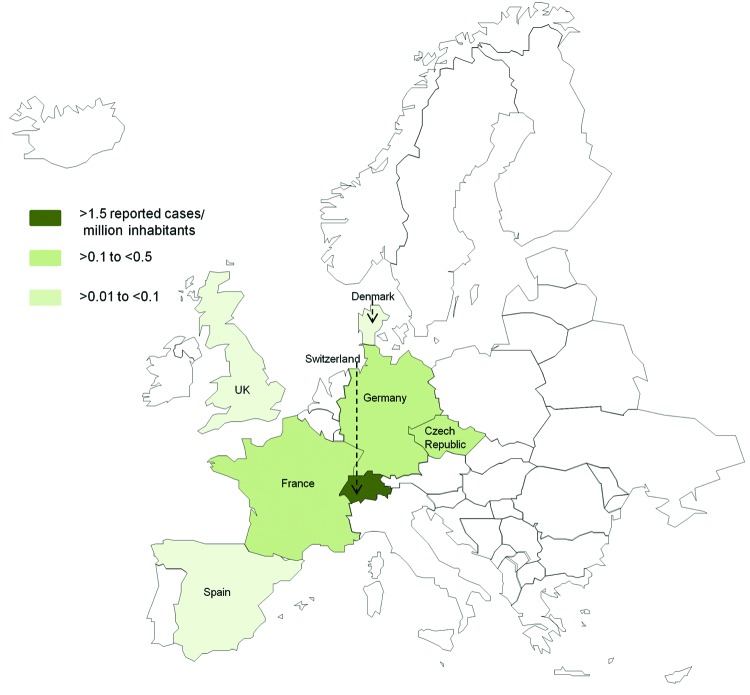
Number of reported cases of *Tropheryma whipplei* endocarditis per 1 million inhabitants in each country of Europe (www.statistiques-mondiales.com/union_europeenne.htm).

**Figure 4:**
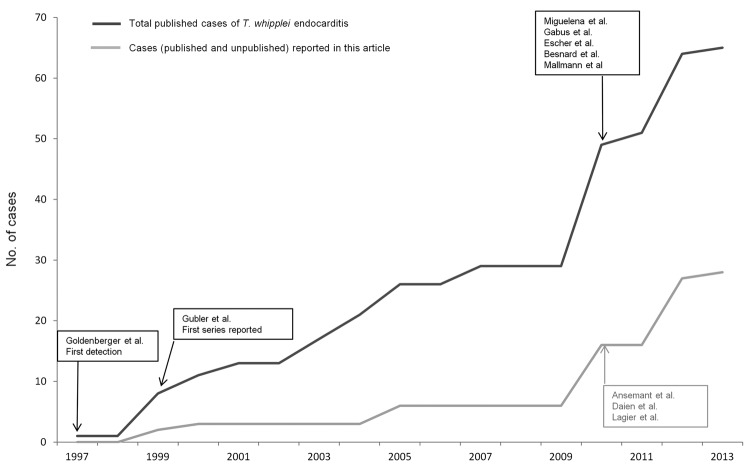
Number of cases of *Tropheryma whipplei* endocarditis reported in the literature since the first detection of this condition in 1997. Cases in 2013 are reported through April.

After checking for repeated reporting, we found 49 patients who met our criteria for *T. whipplei* endocarditis reported in the literature (Technical Appendix Table 4); 7 (14.5%) were female (*3*,*4*,*7*–*11*,*22*–*39*). The patients were predominantly from Germany (15 [30.6%]) and Switzerland (12 [24.5%]). Figure 3 shows the number of reported cases of *T. whipplei* endocarditis per million inhabitants in Europe. The number of cases reported in the literature since 2010 has dramatically increased (Figure 4).

Among the cases reported in the literature, fever was scarcely observed (8/33, 24.2%), but vegetations (28/33, 84.8%) and involvement of the aortic valve (29/48, 60.4%) were frequent. The clinical manifestations were mainly heart failure (25/35, 71.4%), acute ischemic stroke (9/35, 25.7%), and peripheral arterial embolism (4/35, 11.4%). Arthralgia was observed significantly less frequently among patients reported in the literature (15/37, 40.5%) than among the patients we report (75%, p = 0.01). However, if the 14 patients from the recently published series from Germany (*7*) are excluded from the analysis, this difference is not significant (14/23, 60.9%; p = 0.4). The percentage of patients with a history of valvular heart disease was similar among the patients reported here (32%) and the patients reported in the literature (12/33, 36.4%; p = 0.9), but this analysis excludes the series from Germany. The patients in the Germany study experienced significantly more valvular heart disease before diagnosis with endocarditis (13/15, 87%; p = 0.002). The diagnosis was performed by analyzing the removed heart valve for all but 2 of these patients (patients 25 and 33). The clinical manifestations for 2 patients were mainly weight loss, not cardiac disease. The diagnosis for patient 25 was made by a positive PCR on blood and pleural effusion and for patient 39 by a positive PCR from a duodenal biopsy sample. According to the current modified Duke criteria (*20*), before the examination of the heart valve specimens, only 2 (4.25%) patients met the criteria for definite endocarditis (Technical Appendix Table 1). 

Data regarding treatment were available for 45 patients (Technical Appendix Table 4). A total of 43 patients received antimicrobial drugs; at least 15 compounds were used. The most common treatment was trimethoprim–sulfamethoxazole (34 patients); 10 of these patients had previously received ceftriaxone for 2 weeks. The maximum duration of treatment was 2 years; 24 patients received treatment for 1 year. Death was reported for 8 (21%) of 38 patients.

## Discussion

Although the first description of *T. whipplei* endocarditis was made 15 years ago, diagnosing this disease remains difficult because clinical signs are often those of cardiac disease rather than infection. The first case was detected by chance when a broad-spectrum PCR was systematically applied to heart valve specimens (*3*). However, the diagnosis of *T. whipplei* endocarditis is still the result of chance because there are no diagnostic criteria. Thus, diagnosis is still made after 16S rRNA PCR of a removed valve.

The incidence of a disease depends of 3 parameters: physician vigilance, available diagnostic tools, and the true incidence. A team in Switzerland was the first to apply systematic broad-spectrum molecular diagnostics to heart valves (*3*). Although the efforts of that team might explain the high number of reported cases in Switzerland, studies in Marseille, France, that used the same technique did not detect *T. whipplei* in heart valves (*40*). In Germany, several physicians have been interested in Whipple disease for a long time, resulting in the development of new tools (*1*,*32*). In the Rhone-Alpes area of France, physicians have been interested in Whipple disease for several years, resulting in increased attention to *T. whipplei (**26**)*. 

With regard to the global effects of *T. whipplei* endocarditis, there seems to be a geographic gradient with higher incidence in eastern-central France, Switzerland, and Germany. Because 16S rRNA PCR is used in many areas to test for blood culture–negative endocarditis, which would enable detection of *T. whipplei* in heart valves, and significant differences in incidence rates exist, a potential bias seems unlikely (*6*,*12*). Whipple disease reportedly occurs mainly in white persons (*1*). Genotyping shows that a same strain of *T. whipplei* can be involved in chronic infections, acute infections, and chronic carriage. In addition, *T. whipplei* strains are heterogenic; thus, a patient could be colonized multiple times by a new strain (*14*). These data argue for the presence of specific host defects in patients with chronic infections. These defects could be linked to genetic factors that could explain the geographic distribution.

Even in the absence of diagnostic criteria, the reports of ≈50 cases in the literature enabled us to propose several characteristics that might help clinicians recognize potential *T. whipplei* endocarditis. This disease occurs mainly in white men who are ≈50 years of age with cardiac manifestations including heart failure, acute ischemic stroke, and peripheral arterial embolism. These patients might have complained about arthralgia for several years and might have recently received immunosuppressants (*4*,*7*,*28*,*33*,*34*). Arthralgia was not frequently reported among patients in the Germany series (*7*) but was reported as a more prominent symptom by others (*4*,*9*,*26*,*29*,*31*,*33*,*37*,*38*). Arthralgia is sometimes subtle and noticed only after a careful clinical investigation (*37*). Because we have never received articular specimens from these patients, we do not know whether the joints are reactive or correspond to a second localization of *T. whipplei.* Of note, however, these arthralgias are highly sensitive to antimicrobial drugs. Overall, middle-age and older men with subacute endocarditis and no fever or low-grade fever should be asked about the presence of arthralgia because the combination of endocarditis and arthralgia suggests *T. whipplei* infection.

For now, diagnosis of *T. whipplei* endocarditis is made late, performed by molecular analysis of surgically obtained heart valves; specific repeat PCR is used because broad-spectrum PCR might lack sensitivity (*40*). Serologic assays only distinguish between classic Whipple disease and gastrointestinal carriage (*17*). Screening of saliva and fecal specimen has poor predictive value for diagnosis. The diagnostic situation is not satisfactory, but diagnostic improvements are challenging. Only optimization of molecular techniques and culture will enable diagnosis before heart valve analysis. Currently, 16S rRNA amplification performed on blood specimens lacks sensitivity (*6*). Specific repeat PCR is more sensitive (*13*), enabling diagnosis of 31.2% of the patients reported here. In Marseille, for cases of blood culture–negative endocarditis, we systematically apply specific repeat PCR on blood specimens; this protocol enables us to make the diagnosis before heart valve removal (*6*). We suggest adding performance of repeat PCR for *T. whipplei* on blood specimens as a major criterion in the Duke classification for endocarditis, as PCR or serologic testing for *C. burnetii* have been added (*20*). The application of this criterion for patients who have benefited from molecular analysis of blood specimens significantly increases the definitive diagnosis of endocarditis (1/18 vs 6/18; p = 0.03) before the heart valve analysis. In the future, blood specimens from patients with blood culture–negative endocarditis should be also inoculated systematically on specific media. For patients for whom echocardiography is not informative, preliminary data have shown that positron emission tomography and computed tomography show promise, mainly for the detection of silent peripheral embolic events and infectious metastases (*21*).

There is no standard treatment for *T. whipplei* endocarditis. Our series represents a large study with a standardized treatment strategy and follow-up. On the basis of drug sensitivity data, reported resistance of *T. whipplei* to trimethoprim–sulfamethoxazole, and prior experience, a combination of doxycycline and hydroxychloroquine was used. A 12- to 18-month treatment strategy and analysis of the drug concentrations every 3 months seem reasonable. Patients must be forewarned about the risk for photosensitivity when taking doxycycline. All patients in our series have benefitted from heart valve removal; but in the future, to make the diagnosis before heart valve surgery is performed, we advise following the current recommendations for the surgical indications in infective endocarditis (*21*). Even if the approach lacks sensitivity, for patient follow-up, we suggest checking for the presence of *T. whipplei* in the saliva, fecal samples, and blood 2 months after the end of treatment. Subsequent analysis should be performed every 6 months for 2 years and every year for the life of the patient. Echocardiography should be performed yearly to detect relapses. We decided to treat *T. whipplei* recolonization, but we do not know if this measure is necessary.

*T. whipplei* endocarditis differs from classic Whipple disease. Classic Whipple disease involves most organs. Its diagnosis is based on the presence of *T. whipplei*–infected macrophages in intestinal tissues. *T. whipplei* endocarditis is an infection and not a potential cardiovalvular colonization with the bacterium because *T. whipplei* is the only infectious agent detected in heart valves, surrounded by an inflammatory process, and inside the macrophages. For white men >40 years of age with subacute endocarditis and arthralgia, *T. whipplei* infection should be suspected and the organism searched for in blood specimens by using specific repeat PCR and axenic culture, sampled in EDTA and heparin tubes, respectively.

Technical Appendix Classification of the patients with *Tropheryma whipplei* endocarditis; proportion and geographic distribution of *Tropheryma whipplei* endocarditis cases; PCR and histological results for samples from 28 patients with *Tropheryma. *whipplei endocarditis; and characteristics of patients with endocarditis according to literature review.
