# Supplementary material for: Tropheryma whipplei Endocarditis
Source: Emerg Infect Dis. 2013 Nov;19(11):1721–30. doi: 10.3201/eid1911.121356 (PMC3837638; doi:10.3201/eid1911.121356)
Supplement: Technical Appendix — Classification of the patients with Tropheryma whipplei endocarditis; proportion and geographic distribution of Tropheryma whipplei endocarditis cases; PCR and histological results for samples from 28 patients with Tropheryma. whipplei endocarditis; and characteristics of patients with endocarditis according to literature review. [file 12-1356-Techapp-s1.pdf]

Article DOI: http://dx.doi.org/10.3201/eid1911.121356

Tropheryma whipplei Endocarditis

Technical Appendix

Technical Appendix Table 1. Classification of the patients with *Tropheryma whipplei* endocarditis according to the Duke criteria and with the inclusion of positive *T. whipplei* PCR analysis on blood specimens as a major criterion*

	Major criteria		Minor criteria					Duke		
Patient (Ref)	Blood culture	Echocar-diography	Cardiac history	Fever	Microbiological evidence	Vascular phenomena	Immunologic phenomena	Current criteria	Tw blood PCR	Modified according to PCR
Our series										
1	A	Pr	A	A	A	Y	A	P	Neg	P
2	A	A	A	Pr	A	A	A	R	Neg	R
3	A	Pr	A	Pr	A	A	A	P	Pos	D
4	A	Pr	Pr	Pr	A	Pr	A	D	Neg	D
5	A	Pr	A	Pr	A	A	A	P	-	-
6	A	A	A	A	A	A	A	R	-	-
7	A	Pr	A	A	A	A	A	R	Neg	R
8	A	Pr	A	A	A	Pr	A	P	-	-
9	A	Pr	Pr	A	A	A	A	P	-	-
10	A	Pr	A	A	A	A	A	E	-	-
11	A	Pr	A	A	A	A	A	E	-	-
12	A	Pr	Pr	Pr	A	A	A	P	Pos	D
13	A	Pr	A	A	A	A	A	R	-	-
14	A	A	A	Pr	A	Pr	A	R	Neg	R
15	A	Pr	A	Pr	A	A	A	P	Neg	P
16	A	Pr	Pr	Pr	A	A	A	P	Neg	P
17	A	Pr	A	Pr	A	A	A	P	Neg	P
18	A	Pr	Pr	Pr	A	A	A	P	Neg	P
19	A	A	A	Pr	A	A	A	R	Neg	E
20	A	A	A	A	A	Pr	A	R	Pos	D
21	A	Pr	A	A	A	Pr	A	P	Pos	D
22	A	Pr	Pr	A	A	Pr	A	P	-	-
23	A	Pr	A	A	A	A	A	R	-	-
24	A	Pr	Pr	A	A	Pr	A	P	Pos	D
25	A	Pr	A	A	A	A	A	P	Neg	P
26	A	Pr	Pr	A	A	A	A	P	-	-
27	A	Pr	Pr	A	A	A	Pr	P	-	-
28	A	Pr	Pr	A	A	Pr	A	P	-	-
Literature										
1 (*1*)	A	Pr	Pr	A	A	Pr	A	P	-	-
2 (*1*)	A	Pr	Pr	A	A	Pr	A	P	-	-
3 (*1*)	A	-	-	-	A	-	-	*	-	-
4 (*1*)	A	-	-	-	A	-	-	*	-	-
5 (*1*)	A	-	-	-	A	-	-	*	-	-
6 (*1*)	A	-	-	-	A	-	-	*	-	-
7 (*1,2*)	A	-	-	-	A	-	-	*	-	-
8 (*1*)	A	-	-	-	A	-	-	*	-	-
9 (*1*)	A	-	-	-	A	-	-	*	-	-
10 (*1*)	A	-	-	-	A	-	-	*	-	-
11 (*1*)	A	-	-	-	A	-	-	*	-	-
12 (*1*)	A	-	-	-	A	-	-	*	-	-
13 (*1*)	A	-	-	-	A	-	-	*	-	-
14 (*1*)	A	-	-	-	A	-	-	*	-	-
15 (*3*)	A	Pr	A	-	A	Pr	A	P	-	-
16 (*4*)	A	Pr	A	A	A	A	A	R	-	-
17 (*5*)	A	A	Pr	A	A	A	A	R	-	-
18 (*6*)	A	Pr	Pr	A	A	A	A	P	-	-
19 (*6*)	A	Pr	Pr	Pr	A	A	A	P	-	-
20 (*6*)	A	Pr	A	Pr	A	A	A	P	-	-
21 (*7*)	A	Pr	A	A	A	Pr	A	P	-	-

22 (*7*)	A	Pr	A	A	A	A	A	R	-	-
23 (*7*)	A	Pr	A	A	A	A	A	R	-	-
24 (*7*)	A	Pr	Pr	A	A	Pr	A	P	-	-
25 (*8*)	A	Pr	A	A	A	A	A	R	Pos	D
26 (*9*)	A	Pr	A	A	A	A	A	R	-	-
27 (*10*)	A	Pr	A	Pr	A	A	A	P	-	-
28 (*10*)	A	Pr	A	A	A	A	A	R	-	-
29 (*11*)	A	Pr	Pr	A	A	A	A	P	-	-
30 (*12*)	A	Pr	A	Pr	A	A	A	P	Neg	P
31 (*13*)	A	Pr	Pr	Pr	A	Pr	A	D	-	-
32 (*14*)	A	Pr	Pr	A	A	A	A	P	-	-
33 (*15*)	A	Pr	A	Pr	A	A	A	R	-	-
34 (*16*)	A	Pr	Pr	A	A	A	A	P	-	-
35 (*17*)	A	Pr	A	Pr	A	Pr	A	P	-	-
36 (*18*	A	A	A	A	A	A	A	R	-	-
37 (*19*)	A	Pr	Pr	A	A	A	A	P	-	-
38 (*19*)	A	Pr	Pr	A	A	Pr	A	P	-	-
39 (*20*)	A	Pr	A	-	A	A	A	R or P	-	-
40 (*21*)	A	Pr	A	A	A	Pr	A	P	-	-
41 (*22*)	A	A	Pr	A	A	A	A	R	-	-
42 (*23*)	A	A	A	A	A	A	A	R	-	-
43 (*23*)	A	Pr	A	A	A	A	A	R	-	-
44 (*23*)	A	Pr	A	A	A	A	A	R	-	-
45 (*23*)	A	Pr	A	A	A	Pr	A	P	-	-
46 (*24*)	A	Pr	A	Pr	A	Pr	A	P	-	-
47 (*25*)	A	-	-	-	A	-	-	-	-	-
48 (*26*)	A	Pr	Pr	A	A	A	A	P	-	-
49 (*27*)	NA	NA	NA	NA	NA	NA	NA	NA	NA	NA

*Ref, reference; A, absence of the criterion, Pr, presence of the criterion; *, Only one patient among this series fulfilled the Duke criteria for the diagnosis of endocarditis before cardiac valve explantation; -, Not available or Not applicable; P, Possible; D, Definite; R; Rejected.

Technical Appendix Table 2. Proportion and geographic distribution of *Tropheryma whipplei* endocarditis from 702 French patients from the series of Fournier *et al*. (*28*) with blood culture negative endocarditis referred to our center for diagnostic purposes from May 2001 to September 2009*

French area	Number of *T. whipplei* endocarditis (%)	Number of patients tested
Metropolitan France		
Alsace	0	4
Aquitaine	0	11
Auvergne	0	7
Basse-Normandie	0	3
Bourgogne	0	2
Bretagne	0	9
Centre	0	47
Champagne-Ardennes	0	3
Corse	0	2
Franche-Comté	0	6
Haute-Normandie	0	5
Île-de-France	0	92
Languedoc-Roussillon	1 (7.1%)	14
Limousin	-	-
Lorraine	0	7
Midi-Pyrénées	0	18
Nord - Pas-de-Calais	0	10
Pays de la Loire	4 (15.4%)	26
Picardie	1 (8.33%)	12
Poitou-Charentes	1 (2.6%)	38
Provence Alpes Côtes d'Azur (PACA)	0	269
Rhône-Alpes	9 (8.5%)	106
French overseas administrative departments		
Guadeloupe	0	1
Martinique	-	-
Guyane	0	7
Réunion	0	3

*Main significant differences are observed between Rhône-Alpes area and Centre, Île-de-France, and PACA areas ($p = 0.03$, 0.03, and $<10^{-3}$, respectively) and between Pays de la Loire area and Centre, Île-de-France, and PACA areas ($p = 0.01$, 0.001, and $<10^{-3}$, respectively).

Appendix Table 3. PCR and histological results for samples from 28 patients with *Tropheryma. whipplei* endocarditis*

Patient	Leukocytosis	Hypereosinophilia	Increasing CRP	Saliva sample	Stool sample	Blood sample	CSF	Small-bowel biopsy[1]	Cardiac valve
1	Y	Y	Y	PCR-	PCR-	PCR-	-	-	PCR+/PAS+/IHC+/Cult+
2	N	N	Y	PCR-	-	PCR-	-	-	PCR+/PAS+/IHC+/Cult+
3	NA	NA	NA	PCR-	PCR+	PCR+/Cult-	PCR-	PAS-/IHC-/PCR-	PCR+/PAS+/IHC+/Cult-
4	NA	NA	Y	PCR-	PCR-	PCR-	PCR-	PAS-/IHC-/PCR-	PCR+/PAS+/IHC+/Cult-
5	Y	NA	Y	-	-	-	-	PAS-/IHC-/PCR-	PCR+/PAS+/IHC+/Cult-
6	N	N	NA	-	-	-	-	-	PCR+/PAS+/IHC+/Cult-
7	NA	NA	NA	PCR-	PCR-	PCR-	-	-	PCR+/Cult+
8	NA	NA	NA	PCR-	PCR-	-	PCR-	-	PCR+/Cult-
9	NA	NA	NA	-	-	-	-	-	PCR+/PAS+/IHC+/Cult+
10	NA	NA	NA	-	-	-	-	PAS-/IHC-/PCR-	PCR+/PAS+/IHC+
11	NA	NA	NA	-	-	-	-	PAS-/IHC-/PCR-	PCR+/PAS+/IHC+/Cult-
12	N	N	Y	PCR-	PCR-	PCR+/Cult-	-	PAS-/IHC-/PCR-	PCR+/PAS+/IHC+/Cult[2]
13	N	N	Y	-	-	-	-	PAS-	PCR+
14	NA	NA	Y	PCR-	-	PCR-/Cult-	-	PCR-/PAS-/IHC-	PCR+/Cult-
15	N	N	Y	PCR-	PCR-	PCR-	PCR-	-	PCR+/PAS+/IHC+
16	Y	N	Y	PCR-	PCR-	PCR-	PCR-	PAS-	PCR+/PAS+/IHC+
17	N	NA	Y	PCR-	PCR-	PCR-	PCR-	PAS-/IHC-	PCR+
18	NA	NA	N	PCR-	PCR-	PCR-	PCR-	PAS-/IHC-	PCR+/PAS+/IHC+/Cult+
19	N	N	Y	PCR+	PCR+	PCR-	-	PAS-/IHC-/PCR+	PCR+/PAS+/IHC+/Cult-
20	NA	NA	N	PCR-	PCR-	PCR+/Cult+	PCR-	PAS-/IHC-/PCR-	PCR+/Cult+
21	N	N	Y	-	-	PCR+/Cult-	-	PAS-/IHC-/PCR-	PCR+/PAS+/IHC+/Cult-
22	N	Y	Y	PCR-	-	-	-	PAS-/IHC-/PCR-	PCR+/PAS+/IHC+/Cult-
23	N	NA	N	-	-	-	-	PAS-/IHC-/PCR-	PCR+/PAS+/IHC+/Cult-
24	N	N	Y	-	-	PCR+/Cult+	-	PAS-/IHC-/PCR-	PCR+/PAS+/IHC+/Cult-
25	Y	NA	Y	-	-	PCR-/Cult-	-	PAS-/IHC-/PCR-	PCR+/PAS+/IHC+/Cult-
26	NA	NA	N	-	-	-	-	-	PAS+/IHC+/Cult+
27	Y	NA	Y	-	-	-	-	-	PCR+/PAS+/IHC+
28	N	NA	Y	-	-	-	-	PAS-/PCR-	PCR+/PAS+/IHC+

*Y, Yes; N, No; NA, Not available; -, Not Done; CRP, C-reactive protein; CSF, cerebrospinal fluid; PCR-, negative PCR; PCR+, positive PCR; Cult-, Culture negative; Cult+, Culture positive; PAS-, Negative PAS-staining; PAS+, Positive PAS-staining; IHC-, Specific immunohistochemistry negative; IHC+, Specific immunohistochemistry positive; [1]Including duodenum (19 patients), jejunum (8 patients), and ileum (7 patients); [2]A culture of *T. whipplei* has been obtained but the strain has not yet been established.

Technical Appendix Table 4. Characteristics of *Trophyrema whipplei* endocarditis in the literature review (negative PAS-staining duodeno-jejunal biopsy or, at least, without diarrhea)*

Patient (Ref)	Country	Sex	Age, y	Cardiac history	Presenting symptoms	Arthralgia	Fever	SBB	Involved valve	Veg°	Cardiac valve analysis	Antibiotic with duration when available	Outcome (follow-up duration when available)
1 (*1*)	Germany	M	69	Ao regurgitation	HF + stroke	N	N	PAS-	Ao	N	PCR+/PAS+	SXT 1 y	Well, death
2 (*1*)	Germany	M	76	Ao bioprosthesis	HF + stroke	Y	N	-	Ao	N	PCR+/PAS+/IHC+	Cip, SXT 1 y	Lost
3 (*1*)	Germany	M	65	NA	NA	N	NA	-	Mi	NA	PCR+/PAS+/IHC+	C 2 w, SXT 1 y	Well (6 y)
4 (*1*)	Germany	F	60	NA	NA	N	NA	-	Ao	NA	PCR+/PAS+	C 2 w, SXT 1 y	Well (6 y)
5 (*1*)	Germany	M	69	NA	NA	N	NA	-	Ao	NA	PCR+	C 2 w, SXT 1 y	Well, death
6 (*1*)	Germany	M	72	NA	NA	N	NA	-	Ao	NA	PCR+	None	Well (4 y)
7 (*1,2*)	Germany	F	77	NA	NA	N	NA	-	Ao	NA	PCR+/PAS+/IHC+	None	Death
8 (*1*)	Germany	M	63	NA	NA	N	NA	-	Ao	NA	PCR+/PAS+/IHC+	Various antibiotics	Death
9 (*1*)	Germany	M	72	NA	NA	N	NA	-	Ao	NA	PCR+/PAS+/IHC+	C 2 w, D 3 m, SXT 1 y	Well (4 y)
10 (*1*)	Germany	M	46	NA	NA	N	NA	-	Ao	NA	PCR+	C 4 w	Well (3 y)
11 (*1*)	Germany	M	69	NA	NA	N	NA	-	Ao	NA	PCR+/PAS+/IHC+	G 2 w, Blactams, SXT 1 y	Death
12 (*1*)	Germany	F	57	NA	NA	N	NA	-	Mi	NA	PCR+/PAS+	P, V, V+Fosfo 6 w before and after surgery	Well (4 y)
13 (*1*)	Germany	M	56	NA	NA	N	NA	-	Ao	NA	PCR+/PAS+/IHC+	Before surgery: P+G 2 w, P 12 w After surgery: V+P, P 6 w	Lost
14 (*1*)	Germany	M	59	NA	NA	N	NA	-	Ao	NA	PCR+	C 2 w, SXT 1 y	Well (3 y)
15 (*3*)	Canada	M	48	N	HF + stroke	N	NA	-	Ao	Y	PCR+/PAS+	C +V 6 w, C +SXT 6 w, SXT 14 m	Well (14 m)
16 (*4*)	UK	M	66	N	HF	Y	N	-	Ao + Mi	Y	PCR+	Cip+Flucoxacillin+G, V+G+R, C 4 w SXT 12 m	Well
17 (*5*)	France	M	66	Mi prolapsed	HF	NA	N	-	Mi	Y	PCR+/PAS+	D+H 18 m	Well
18 (*6*)	France	M	72	Ao+Mi Bioprostheses	HF	NA	N	-	Ao + Mi	Y	PCR+	C 2 w, SXT 1 y	Well (4 y)
19 (*6*)	France	M	59	Mi regurgitation	HF	NA	Y	-	Mi	Y	PCR +	C 2 w, SXT 1 y	Well (1 y)
20 (*6*)	France	M	56	N	HF	NA	Y	-	Ao	Y	PCR+	C+G 2 w, SXT 1 y	Well (5 m) Death
21 (*7*)	Switzerland	M	54	N	Peripheral embolism	Y	N	-	Ao	Y	PCR+/PAS+	C+G 4 w, SXT 1 y	Well (4 ½ y)
22 (*7*)	Switzerland	M	78	Coronary heart disease	HF	N	N	-	Ao	Y	PCR+/-	C+G 2 w, SXT 1 y	Well (4 ½ y)
23 (*7*)	Switzerland	M	70	N	HF	N	N	-	Mi	Y	PCR+/PAS+	C 2 w, SXT 1 y	Well (2 y)
24 (*7*)	Switzerland	M	76	Heart insufficiency	Stroke	N	N	-	Mi	Y	PCR+/PAS-	C 2 w, SXT 1 y	Well (2 y)
25 (*8*)	Switzerland	M	50	N	Weakness, weight loss	N	N	-	Tri	Y	-	AmxClav, C, D+H+SXT	Well (1 y)
26 (*9*)	Spain	F	57	N	HF	Y	N	-	Ao	Y	PCR+	C 1 w, SXT 18 m Ongoing	Well
27 (*10*)	Denmark	M	51	N	HF	Y	Y	PAS-	Ao	Y	PCR+/PAS+	Amp+G+M 3 w, Cip+M+Dif 10 d, Mer+Cip+M+Dif 5 w,	Death

Patient (Ref)	Country	Sex	Age, y	Cardiac history	Presenting symptoms	Arthralgia	Fever	SBB	Involved valve	Veg°	Cardiac valve analysis	Antibiotic with duration when available	Outcome (follow-up duration when available)
												SXT 1 w	
28 (*10*)	Denmark	M	67	N	HF	Y	N	-	Ao	Y	PCR+/PAS+	P+G 4 w, SXT 1 y	Well (9 m)
29 (*11*)	UK	M	38	BAV	HF	Y	N	-	Ao	Y	PCR+/PAS+	NA	NA
30 (*12*)	Spain	M	51	N	HF	N	Y	-	Ao	Y	PCR+/PAS+	V+G, V+G+C, SXT 1 y	Well (1 y)
31 (*13*)	Czech Republic	M	33	BAV	Stroke + Peripheral embolism	Y	Y	PAS-	Mi	Y	PCR+	V 3 w, Cip 6 w, C 3 w, T 6 m	Well (6 m)
32 (*14*)	UK	M	58	Cardiomyopathy	HF	NA	N	PAS-	Ao	Y	PCR+/PAS-	Antibiotics	Death
33 (*15*)	France	M	61	N	Weight loss	NA	Y	PAS- /PCR+	Ao	Y	-	Amx+G 3 w, SXT 13 m	Well (13 m)
34 (*16*)	Germany	F	80	Ao bioprosthesis	HF	NA	N	PAS- /PCR-	Ao	N	PCR+	C 2 w, SXT 1 y	Well (1 y)
35 (*17*)	Tunisia	M	62	N	Stroke + Peripheral embolism	NA	Y	PAS- /PCR-	Ao	Y	PCR+	Amx+G, Cefo 3 w, SXT 1 y	Well (2 ½ m)
36 (*18*)	Switzerland	NA	NA	N	HF	Y	N	-	Ao	N	PCR+	NA	NA
37 (*19*)	Canada	M	65	BAV	HF	N	N	-	Ao	Y	PCR+	V, SXT 1 y	NA
38 (*19*)	Canada	M	51	BAV	Peripheral embolism	N	N	-	Ao	Y	PCR+	V 4 w, SXT 1 y	NA
39 (*20*)	Czech Republic	M	68	Coronary heart disease	Valve dysfunction	NA	NA	-	Ao	NA	PCR+	NA	NA
40 (*21*)	Switzerland	F	51	N	Stroke	Y	N	PAS-/ PCR-	Mi	Y	PCR+/PAS-	SXT 1 y	Well (1 y)
41 (*22*)	USA	M	78	Heart insufficiency	HF	N	N	PAS-	Ao	N	PCR+/PAS+	V+G	NA
42 (*23*)	Switzerland	M	64	N	HF	Y	N	PAS-/ PCR-	Ao	N	PCR+	P+G 6 w, SXT 2 y	Well (36 m)
43 (*23*)	Switzerland	M	53	N	HF	Y	N	PAS-/ PCR+	Ao	Y	PCR+/PAS-	C 6 w, SXT 1 y	Well (32 m)
44 (*23*)	Switzerland	M	55	N	HF	Y	N	PAS-/ PCR-	Ao +Mi	Y	PCR +	SXT ongoing	Well (1 y)
45 (*23*)	Switzerland	F	55	N	Stroke	Y	N	PAS-/ PCR-	Mi	Y	PCR+/PAS+	C 6 w, SXT ongoing	Well (6 m)
46 (*24*)	USA	M	43	N	HF + stroke	NA	Y	PAS-	Ao +Mi	Y	PCR+/PAS+	SXT ongoing	Well (7 m)
47 (*25*)	Switzerland	NA	NA	NA	NA	NA	NA	-	Mi	NA	PCR+	NA	NA
48 (*26*)	UK	M	50	BAV	HF	Y	N	-	Ao +Mi	Y	PCR+/PAS+	C 2 w, SXT ongoing	Well (9 m)
49 (*27*)	USA	NA	NA	NA	NA	NA	NA	NA	NA	NA	PCR MS+	NA	NA

*Ref, Reference; SBB, Small-bowel biopsy; Veg°, Vegetation; BAV, Biscupsid Aortic Valve; -, Not done, NA, Not available; Y, yes; No, no
Peripheral embolism, Peripheral arterial embolism; Stroke, Acute ischemic stroke; PCR MS, PCR electrospray ionization Mass spectrometry
Amp, Ampicillin; Amx, Amoxicillin; AmxClav, Amoxicillin and Clavulanic acid; Cefo, Cefotaxime; Cip, Ciprofloxacin; D, Doxycycline; G, Gentamicin; H, Hydroxychloroquine; M, metronidazole; Mer, meropenem; P, Penicillin; R, Rifampicin; SXT, trimethoprim and sulfamethoxazole; T, trimethoprim; V, vancomycine; y, year(s); m, month(s), w, week(s).

References

1. Geissdörfer W, Moos V, Moter A, Loddenkemper C, Jansen A, Tandler R, et al. High frequency of *Tropheryma whipplei* in culture-negative endocarditis. J Clin Microbiol. 2012;50:216–22. PubMed http://dx.doi.org/10.1128/JCM.05531-11

2. Mallmann C, Siemoneit S, Schmiedel D, Petrich A, Gescher DM, Halle E, et al. Fluorescence in situ hybridization to improve the diagnosis of endocarditis: a pilot study. Clin Microbiol Infect. 2010;16:767–73. PubMed http://dx.doi.org/10.1111/j.1469-0691.2009.02936.x

3. Chan V, Wang B, Veinot JP, Suh KN, Rose G, Desjardins M, et al. *Tropheryma whipplei* aortic valve endocarditis without systemic Whipple's disease. Int J Infect Dis. 2011;15:e804–6. PubMed http://dx.doi.org/10.1016/j.ijid.2011.05.020

4. Whistance RN, Elfarouki GW, Vohra HA, Livesey SA. A case of *Tropheryma whipplei* infective endocarditis of the aortic and mitral valves in association with psoriatic arthritis and lumbar discitis. J Heart Valve Dis. 2011;20:353–6. PubMed

5. Brondex A, Jobic Y. Infective endocarditis as the only manifestation of Whipple's disease: an atypical presentation. Ann Cardiol Angeiol (Paris). 2012;61:61–3. PubMed http://dx.doi.org/10.1016/j.ancard.2010.12.005

6. Besnard S, Cady A, Flecher E, Fily F, Revest M, Arvieux C, et al. Should we systematically perform central nervous system imaging in patients with Whipple's endocarditis? Am J Med. 2010;123:962.e–4. PubMed http://dx.doi.org/10.1016/j.amjmed.2010.04.030

7. Escher R, Roth S, Droz S, Egli K, Altwegg M, Tauber MG. Endocarditis due to *Tropheryma whipplei:* rapid detection, limited genetic diversity, and long-term clinical outcome in a local experience. Clin Microbiol Infect. 2010;16:1213–22. PubMed http://dx.doi.org/10.1111/j.1469-0691.2009.03038.x

8. Gabus V, Grenak-Degoumois Z, Jeanneret S, Rakotoarimanana R, Greub G, Genne D. *Tropheryma whipplei* tricuspid endocarditis: a case report and review of the literature. J Med Case Rep. 2010;4:245. PubMed http://dx.doi.org/10.1186/1752-1947-4-245

9. Miguelena J, Munoz R, Maseda R, Epeldegui A. Endocarditis due to *Tropheryma whipplei.* Rev Esp Cardiol. 2010;63:250–1. PubMed http://dx.doi.org/10.1016/S0300-8932(10)70052-X

10. Voldstedlund M, Pedersen LN, Baandrup U, Fuursted K. Whipple's disease–a cause of culture-negative endocarditis. Ugeskr Laeger. 2004;166:3731–2. PubMed

11. Williams OM, Nightingale AK, Hartley J. Whipple's disease. N Engl J Med. 2007;356:1479–81. PubMed http://dx.doi.org/10.1056/NEJMc070234

12. Marín M, Muñoz P, Sánchez M, del Rosal M, Alcalá L, Rodríguez-Créixems M, et al. Molecular diagnosis of infective endocarditis by real-time broad-range polymerase chain reaction (PCR) and sequencing directly from heart valve tissue. Medicine (Baltimore). 2007;86:195–202. PubMed http://dx.doi.org/10.1097/MD.0b013e31811f44ec

13. Kolek M, Zaloudikova B, Freiberger T, Brat R. Aortic and mitral valve infective endocarditis caused by *Tropheryma whipplei* and with no gastrointestinal manifestations of Whipple's disease. Klin Mikrobiol Infekc Lek. 2007;13:213–6. PubMed

14. West D, Hutcheon S, Kain R, Reid T, Walton S, Buchan K. Whipple's endocarditis. J R Soc Med. 2005;98:362–4. PubMed http://dx.doi.org/10.1258/jrsm.98.8.362

15. Saba M, Rollot F, Park S, Grimaldi D, Sicard D, Abad S, et al. Whipple disease, initially diagnosed as sarcoidosis. Presse Med. 2005;34:1521–4. PubMed http://dx.doi.org/10.1016/S0755-4982(05)84217-4

16. Dreier J, Szabados F, von Herbay A, Kroger T, Kleesiek K. *Tropheryma whipplei* infection of an acellular porcine heart valve bioprosthesis in a patient who did not have intestinal Whipple's disease. J Clin Microbiol. 2004;42:4487–93. PubMed http://dx.doi.org/10.1128/JCM.42.10.4487-4493.2004

17. Marrakchi C, Abdennadher M, Blin D. Endocarditis caused by *Tropheryma whippelii*. Tunis Med. 2004;82:781–4. PubMed

18. Bosshard PP, Kronenberg A, Zbinden R, Ruef C, Bottger EC, Altwegg M. Etiologic diagnosis of infective endocarditis by broad-range polymerase chain reaction: a 3-year experience. Clin Infect Dis. 2003;37:167–72. PubMed http://dx.doi.org/10.1086/375592

19. Richardson DC, Burrows LL, Korithoski B, Salit IE, Butany J, David TE, et al. *Tropheryma whippelii* as a cause of afebrile culture-negative endocarditis: the evolving spectrum of Whipple's disease. J Infect. 2003;47:170–3. PubMed http://dx.doi.org/10.1016/S0163-4453(03)00015-X

20. Grijalva M, Horvath R, Dendis M, Erny J, Benedik J. Molecular diagnosis of culture negative infective endocarditis: clinical validation in a group of surgically treated patients. Heart. 2003;89:263–8. PubMed http://dx.doi.org/10.1136/heart.89.3.263

21. Naegeli B, Bannwart F, Bertel O. An uncommon cause of recurrent strokes: *Tropheryma whippelii* endocarditis. Stroke. 2000;31:2002–3. PubMed http://dx.doi.org/10.1161/01.STR.31.8.2002

22. Smith MA. Whipple endocarditis without gastrointestinal disease. Ann Intern Med. 2000;132:595. PubMed http://dx.doi.org/10.7326/0003-4819-132-7-200004040-00025

23. Gubler JG, Kuster M, Dutly F, Bannwart F, Krause M, Vögelin HP, et al. Whipple endocarditis without overt gastrointestinal disease: report of four cases. Ann Intern Med. 1999;131:112–6. PubMed http://dx.doi.org/10.7326/0003-4819-131-2-199907200-00007

24. Elkins C, Shuman T, Pirolo J. Cardiac Whipple's disease without digestive symptoms. Ann Thorac Surg. 1999;67:250–1. PubMed http://dx.doi.org/10.1016/S0003-4975(98)01204-1

25. Goldenberger D, Kunzli A, Vogt P, Zbinden R, Altwegg M. Molecular diagnosis of bacterial endocarditis by broad-range PCR amplification and direct sequencing. J Clin Microbiol. 1997;35:2733–9. PubMed

26. Love SM, Morrison L, Appleby C, Modi P. *Tropheryma whipplei* endocarditis without gastrointestinal involvement. Interact Cardiovasc Thorac Surg. 2012;15:161–3. PubMed http://dx.doi.org/10.1093/icvts/ivs116

27. Brinkman CL, Vergidis P, Uhl JR, Pritt BS, Cockerill FR, Steckelberg JM, et al. Polymerase chain reaction-electrospray ionization mass spectrometry for direct detection of pathogens and antimicrobial resistance from heart valves in patients with infective endocarditis. J Clin Microbiol. 2013; Epub ahead of print. PubMed http://dx.doi.org/10.1128/JCM.00304-13

28. Fournier PE, Thuny F, Richet H, Lepidi H, Casalta JP, Arzouni JP, et al. Comprehensive diagnostic strategy for blood culture–negative endocarditis: a prospective study of 819 new cases. Clin Infect Dis. 2010;51:131–40. PubMed http://dx.doi.org/10.1086/653675
